# Development and evaluation of an assessment of the age-appropriateness/inappropriateness of formulations used in children

**DOI:** 10.1007/s11096-022-01478-5

**Published:** 2022-10-08

**Authors:** Jennifer C. Duncan, Louise E. Bracken, Anthony J. Nunn, Matthew Peak, Mark A. Turner

**Affiliations:** 1 Paediatric Medicines Research Unit, Institute in the Park, Alder Hey Children’s NHS Foundation Trust, Liverpool, UK; 2 Department of Women’s and Children’s Health, Institute of Translational Medicine, University of Liverpool, Liverpool Health Partners, Liverpool, UK; 3 Liverpool Women’s NHS Foundation Trust, Liverpool, UK

**Keywords:** Age-appropriate formulations, Children, Off-label prescribing, Unlicensed medicines

## Abstract

**Background:**

Medicines designed for adults may be inappropriate for use in children in terms of strength, dosage form and/or excipient content. There is currently no standardised method of assessing the age-appropriateness of a medicine for paediatric use.

**Aim:**

To develop and test a tool to assess whether a dosage form (formulation) is appropriate for children and estimate the proportion of formulations considered ‘inappropriate’ in a cohort of hospitalised paediatric patients with a chronic illness.

**Method:**

A multi-phase study: patient data collection, tool development, case assessments and tool validation. Inpatients aged 0–17 years at two UK paediatric/neonatal hospitals during data collection periods between January 2015 and March 2016. Written informed consent/assent was obtained. Medicines assessed were new or regularly prescribed to inpatients as part of their routine clinical care. All medicine administration episodes recorded were assessed using the Age-appropriate Formulation tool. The tool was developed by a consensus approach, as a one-page flowchart. Independent case assessments were evaluated in 2019.

**Results:**

In 427 eligible children; 2,199 medicine administration episodes were recorded. Two assessors reviewed 220 episodes in parallel: percentage exact agreement was found to be 91.7% (99/108) and 93.1% (95/102). In total, 259/2,199 (11.8%) medicine administration episodes involved a dosage form categorised as ‘age-inappropriate’.

**Conclusion:**

A novel tool has been developed and internally validated. The tool can identify which medicines would benefit from development of an improved paediatric formulation. It has shown high inter-rater reliability between users. External validation is needed to further assess the tool’s utility in different settings.

**Supplementary Information:**

The online version contains supplementary material available at 10.1007/s11096-022-01478-5.

## Impact statements

- A list of age-inappropriate formulations was identified which could help inform paediatric medicine development by indicating which products need an age-appropriate formulation.
- These assessments provide information to manufacturers about how their product is being administered in hospital practice. Assessment of age-appropriateness is important in product development.
- Assessment of the age-appropriateness of a medicine is an important step when prescribing and dispensing for children, particularly younger children. Clinicians should discuss the most appropriate treatment options available with the child and/or their caregiver to meet the patient’s individual needs.

## Introduction

Medicines prescribed for children have not always been designed with children in mind. Children and young people (CYP) have been called ‘therapeutic orphans’ due to lack of research and commercially obtainable, age-appropriate formulations (AaFs) [1]. Consequently, clinicians often have no choice but to treat children with medicines being used in unapproved ways (off-label, OL) or for unlicensed (UL) medicines to be sourced to fill this gap [2–4]. CYP have specific requirements for medicines distinct from those of adults. A child may lack physical and/or mental ability to use a medicine as intended by the manufacturer. Medicines which are not age-appropriate must be compounded, manipulated, or modified before administration to obtain the required dose and/or facilitate administration [5–7]. Changes to the marketed, quality assured product may lead to safety concerns and reduced efficacy [8–12]. Children deserve access to medicines that have been developed for their individual needs [13, 14]. It is important that medicines can provide accurate dosing for use across a diverse population; dosing requirements can vary 100-fold from birth to adolescence [15, 16].

In this study, an AaF was defined as “An authorised medicine used within the terms of its marketing authorisation (MA) which can be taken/used by the child (either directly or indirectly via a carer) without any issues on administration i.e. overall it was acceptable to that patient and was used as intended” [17]. An administration issue was defined as the need to take a practical action to change or modify the dosage form (DF) supplied to allow or facilitate administration of the prescribed dose. Therefore, AaFs are licensed medicines used in accordance with the MA and acceptable to the patient. Age-inappropriate formulations (AiFs) require manipulation at the point of administration (by caregivers/patients) to allow the intended dose of the DF prescribed to be administered to the patient in an acceptable manner [17].

Reasons for using AiFs may include absence of a suitable product on the market, a purchasing decision based on cost or safety concerns, patient preference or temporary need until a more appropriate medicine can be obtained [18–20].

The extent of the problem and impact on caregivers/patients when suitable formulations are unavailable is largely unknown [21]. Assessment of ‘age-appropriateness’ or whether a medicine is acceptable for children can be “context-independent” (e.g. a DF that can be successfully administered to all three month olds) or “context-dependent” (e.g. a DF that some five years will accept but not all because of taste preferences). To address both aspects of each episode, AaF assessment needs to follow a systematic, validated, structured approach. An AaF tool is primarily designed to guide the pharmaceutical industry and policy makers on which medicines could benefit from development of an AaF. Our tool uses 'real world data' regarding the users (carer/patient) experience of administering the medicine as prescribed whilst others adopt a literature and expert opinion approach [22–24].

### Aim

To develop and test a tool to assess whether a DF (formulation) is appropriate for children and estimate the proportion of formulations considered ‘inappropriate’ in a cohort of hospitalised paediatric patients with a chronic illness.

### Ethics approval

UK NHS Research Ethics Committee (North-West Liverpool East, REC Reference:14/NW/1437, Approval Date:08/12/2014) and the Health Research Authority (HRA), Integrated Research Application System (IRAS) no:164741.

## Method

### Clinical study of medicine administration episodes among paediatric inpatients: the AaF study

The tool was developed using a selection of cases (purposive sampling) gathered in the AaF study. Accordingly, data collection is described first. Data collection provided both a broad and representative corpus of medicine administration episodes (MAEs) for tool development and a separate, but much larger, dataset for the evaluation of MAEs.

### Study design and participants

A prospective, observational study collected MAE data from a UK tertiary paediatric hospital (Alder Hey Children’s NHS FT, (AH)) and neonatal unit (Liverpool Women’s NHS FT, (LWH)) across several recruitment periods, between January 2015 and March 2016 (18 weeks in total). On admission, patients were tracked (until discharged from admission ward) and assigned a unique study ID code. Targeted paediatric speciality wards included cardiology, nephrology, rheumatology, gastroenterology, neonatology, and neurology (ward based “sample of convenience”).

### Consent to participate

Written informed consent was obtained from parent/guardian for children under 16 years; 16–17 years provided own consent; written assent was requested from children over 6 years. Consent allowed the research team to access patient clinical/prescription records needed to assess study eligibility.

### Eligibility

Participants aged 0–17 years, diagnosed with a long-term medical condition (LTMC) and taking at least one medicine at the time of review. Prematurity was included as a LTMC.

### Data collection

Data were gathered by review of clinical records and in person. Details of MAEs were recorded on a case report form (CRF), noting how the intended dose was prepared and administered, including any physical modification of the DF and/or if any adaptation was needed to aid administration; for example, mixing with a drink due to a palatability issue. Product brand/manufacturer details were recorded. The researchers did not directly observe the medicines being prepared or administered.

Each medication review provided a “snapshot” of all medicines to be administered on that day to the study participant. Further consent was obtained for patients readmitted during a subsequent data collection period and a new study ID code assigned.

All pharmaceutical preparations of medicines prescribed were included for assessment including tablets, capsules, oral liquids, inhalers, nebules, dermatologicals, suppositories and injections. Medical devices (e.g. sodium chloride 7% nebules (Nebusal®)) were also screened for suitability. Intravenous fluids, parenteral nutrition, dialysis products, dressings, cleansing agents, cosmetics and food supplements were excluded.

Data were transferred to a spreadsheet (Microsoft Excel 2016, Version 16.0) for each hospital site.

### Development of the tool

**Fig. 1:**
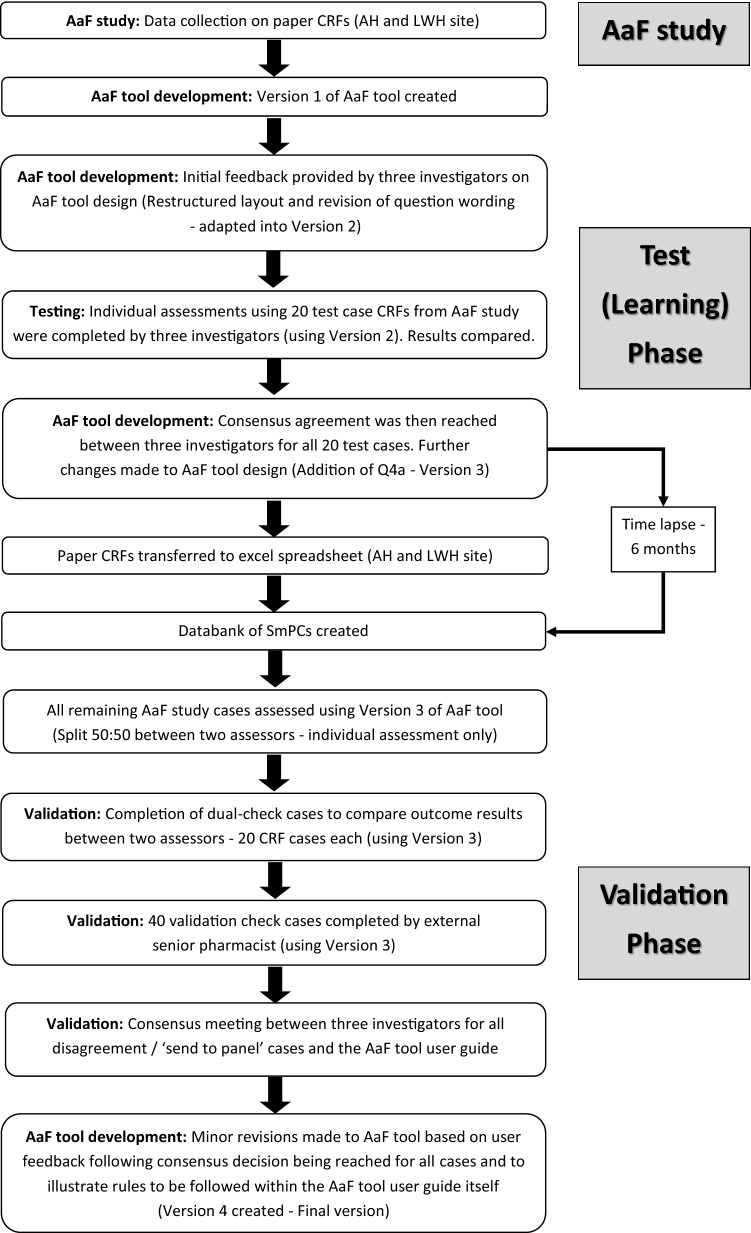
Stages of development of the AaF tool

The AaF tool is a flowchart designed to aid categorisation of medicines in terms of their age-appropriateness. As shown in Fig. 1, the development of the AaF tool occurred over two phases—(I) learning (test stage) and (II) validation.

During the learning phase, three pharmacist investigators independently assessed 20 ‘test cases’ (AH 16/LWH 4). The ‘test cases’ represented a total of 112 MAEs from the AaF study. This was a purposive sample selected to include a variety of DFs, different routes of administration (RoAs) and included MAEs across the full age spectrum (neonates, infants, young child, older child, teenagers, and adolescents) [25, 26]. A comparison of the ‘test case’ age-appropriateness outcomes was made. Any discrepancies were discussed between the three investigators (“The panel”) until consensus agreement was reached on the ‘age-appropriate’ outcome for each of the test case reviews.

**Fig. 2:**
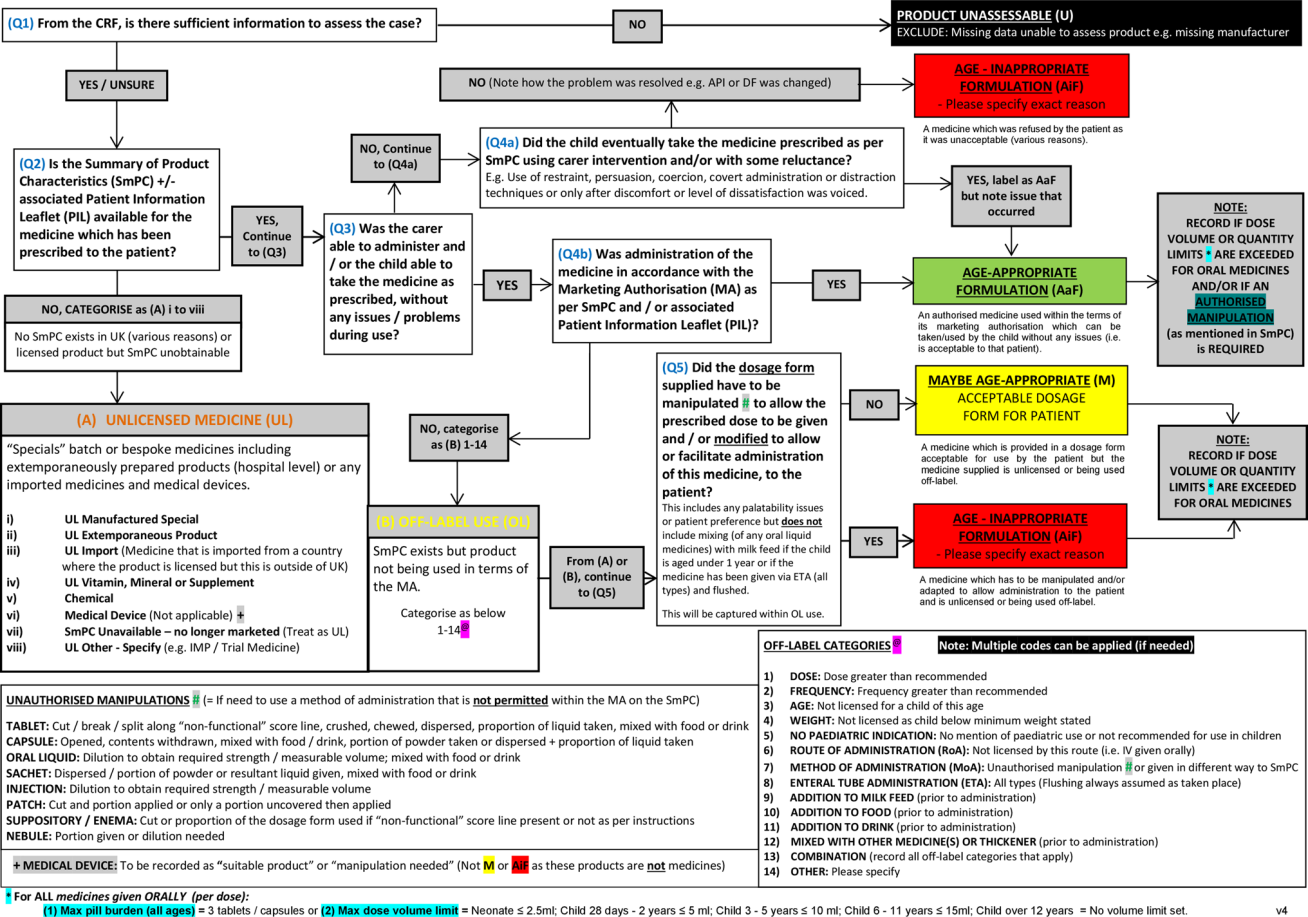
The AaF tool (Final version)

An adapted version of the tool was developed through consensus between the three investigators. Each question (included in the AaF tool) and resulting ‘pathways’ were reviewed by the research team to ensure relevance and that all possible MAE scenarios had been covered. Any questions leading to disagreement between users were adapted appropriately by consensus. The final version of the tool (Fig. 2) consists of five questions, with dichotomous responses to each question.

### Categorisation of age-appropriateness

Using the tool, the assessor assigned one of seven age-appropriate outcome result categories. A further detailed sub-category could also be selected to aid data analysis. The assessor noted any specific reasoning for their decision; for example, if a medicine had been added to food to aid administration. Further detail is provided in Online Resource 1.

### Inter-observer validation

To test for differences between users of the tool, inter-rater reliability (IRR) was measured. For a case to be marked as ‘matched agreement’, the detailed sub-category outcome assigned by each assessor matched exactly (Online Resource 1).

**Fig. 3:**
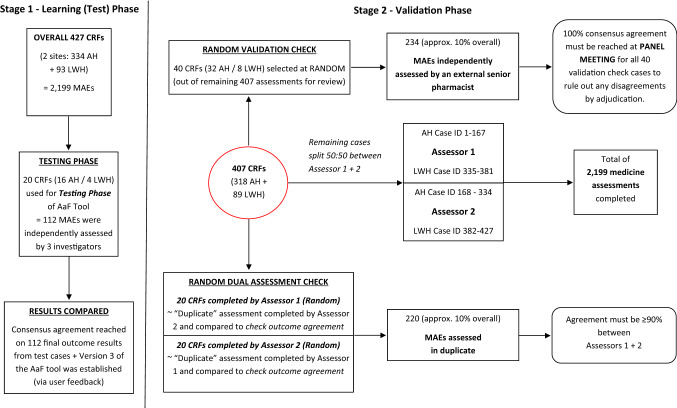
AaF tool development and validation

As seen in Fig. 3, the 20 ‘test cases’ previously reviewed during the initial learning phase were not included in the validation stage. Remaining cases were divided equally between two assessors for independent assessment. Random validation checks between assessors and an external senior pharmacist were undertaken.

### Characterisation of medicine administration episodes

Any medicines prescribed and intended to be given on the day of review (24-h) were recorded for each patient. Patient characteristics included age, gender, weight, attending consultant and LTMC diagnosis. Product information included drug name, strength of active pharmaceutical ingredient (API), DF, manufacturer (including brand name), how the medicines was used/taken, administration device, prescribed dose, frequency, and route. Drug history was verified to determine if the item prescribed was new or taken prior to admission.

### Data analysis

Statistical Package for the Social Science (SPSS), Version 24 for descriptive analysis of data. Each individual MAE was used as the unit of analysis (UoA).

## Results

### Data collection: medicine administration episodes among paediatric inpatients

A total of 610 participants (including 15 readmitted at AH) were consented into the AaF study. 2,199 MAEs were recorded as being successfully administered to 427 children. Infants (28 days to 11 months) were the most common age group evaluated (104/427, 24.4%). Demographic analysis is shown in Table 1.

**Table 1:** Study participant demographics

No. of participants	AH	LWH	Total
Consented (to access medical records)	510	100	610
Eligible	334	93	427
Male	190	59	249 (58.3%)
Number of MAEs (number of patients)	1758 (334)	441 (93)	2199 (427)
Mean, median and range of medicines per patient	5.26; 4; 1–28	4.74; 4; 1–15	
Age groups			
Extremely preterm neonate (< 28 weeks)		8	8
Very preterm neonate (28–< 32 weeks)		31	31
Moderate to late preterm neonate (32–< 37 weeks)		38	38
Term neonate (> 37 weeks+)	37	14	51
Infant (28 days to ≤ 11 months)	102	2	104
Toddler (12–≤ 23 months)	33		33
Young child (2–≤ 5 years)	60		60
Older child (6–≤ 11 years)	49		49
Teenager (12–≤ 15 years)	35		35
Adolescent (16–< 18 years old)	18		18

### Inter-observer agreement

Assessor 1 checked 111 MAE assessments completed by Assessor 2. Assessor 2 checked 109 MAE assessments completed by Assessor 1. Percentage exact agreement (%EA) of MAE assessments was found to be 91.7% (99/108) and 93.1% (95/102) for Assessor 1 and Assessor 2, respectively as 10 MAEs were referred to panel. Overall %EA of 92.4% (194/210) for all 40 ‘dual-check’ cases was found and therefore no further duplicate checks were undertaken to test the reproducibility of assessment outcomes by different reviewers. Assessor 3 (senior pharmacist) checked 234 MAE assessments completed by Assessor 1/2 (or both if duplicated case). 29 MAEs were referred to panel. Assessor 3 had %EA of 83.9% (174/205) against either assessor. Any disagreements found (including all ‘refer to panel’ cases) were adjudicated and resolved by discussion at the panel consensus meeting thus 100% agreement was reached for all 40 ‘dual check’ and 40 ‘validation’ cases reviewed.

### Characterisation of medicine administration episodes

**Fig. 4:**
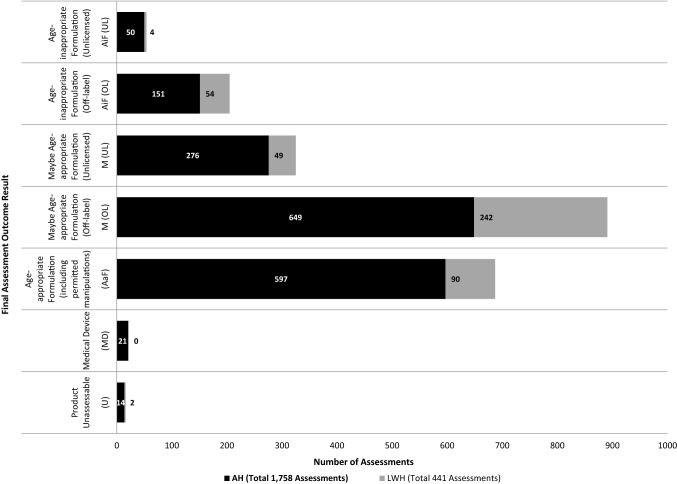
AaF assessment outcome results

Results of the MAE assessments are shown in Fig. 4. In total, 259/2,199 (11.8%) MAEs were found to use AiFs, of which 201/259 (77.6%) were at AH site. At AH 34.0% (597/1,758) MAEs episodes and 20.4% (90/441) MAEs at LWH were classified as AaFs. The prevalence of AiFs detected was inversely proportional to age at each site (Online Resource 2). At AH the main reason for manipulating a medicine was DF issue (39% of 201 MAEs) as in general, a smaller dose was required than was available; followed by need to facilitate administration (35%, 70/201). At LWH, this was found to be 67% for DF issue and 26% to facilitate administration of the 58 MAEs recorded as AiFs.

The greatest proportion of MAEs recorded at both sites involved a licensed medicine being used in an OL manner (AH 46.4% (800/1,723), LWH 67.4% (296/439)). At AH, 7.7% (107/1,397) of all the medicines given did not have a paediatric indication; at LWH, this figure was 4.4% (17/386). Only 20.5% (90/439) of MAEs captured at LWH were recorded as being given in a licensed way, according to the products MA. The number of authorised medicine administrations captured was higher at AH with 597/1,723 (34.6%) being given as intended and in accordance with the products MA. This included any permitted manipulations needed. UL medicine use was found to be 18.9% (326/1,723) and 12.1% (53/439) of all MAEs documented at AH and LWH sites, respectively.

Medicines categorised as AiFs more than once in the study are shown in Table 2. The most common AiFs identified were Melatonin 2 mg Capsules and Phosphate 500 mg Effervescent Tablets at AH and LWH sites, respectively. At AH, a third (65/201, 32.3%) of MAEs subsequently categorised as AiFs involved a solid oral dosage form (SODF) such as tablets/capsules (24 distinct APIs). The greatest proportion of AiFs identified were administered via the oral route (29.4%, 59/201) closely followed by Percutaneous Endoscopic Gastrostomy (PEG) administration (27.4%, 55/201) at AH. At LWH most AiFs were administered parenterally (60.3%, 35/58) followed by administration via orogastric tube (OGT) (17.2%, 10/58).

**Table 2:** AiFs identified in the study (both sites) with a frequency > 1

AiFs-AH	Frequency	AiF reason code	AiFs-LWH	Frequency	AiF reason code
Melatonin 2 mg capsule	21	DF(T)/PF/PP/EA	Phosphate 500 mg (16.1 mmol) effervescent tablet	7	DF(S)/PF
Omeprazole 10 mg MUPs tablet	19	DF(S + T)/PF	Teicoplanin 200 mg powder for solution for injection or infusion	6	DF(S)
Aspirin 75 mg dispersible tablet	16	DF(S)/Pal/PF/PP	Fluconazole 2 mg per ml solution for Infusion	4	DF(S)/EA
Chloral hydrate 500 mg per 5 ml oral liquid	11	LP	Hydrocortisone 100 mg powder for solution for injection or infusion	4	DF(S)
Hyoscine 1.5 mg patch	11	DF(S)	Morphine sulphate 1 mg per ml solution for injection or infusion	4	DF(S)
Furosemide 50 mg per 5 ml SF oral solution	6	Pal/PF/LP	Sodium chloride 3 g in 10 ml (30%) concentrate for solution for infusion	4	DF(S)
Loperamide 2 mg capsule	6	DF(S + T)/PF	Meropenem 500 mg solution for injection or infusion	3	DF(S)/LP
Levothyroxine 25 mcg tablet	5	DF(S + T)/PF	Midazolam 5 mg per ml solution for injection, infusion or rectal use	3	DF(S)
Movicol paediatric plain 6.9 g powder for oral solution	5	DF(S)/PF	Multivitamin drops	3	EA
Spironolactone 50 mg per 5 ml oral suspension	5	Pal/PF/LP	Enoxaparin 20 mg in 0.2 ml pre-filled syringe injection	2	DF(S)
Amiodarone 100 mg tablet	4	DF(S + T)	Folic acid 2.5 mg per 5 ml oral solution	2	EA
Dinoprostone 1 mg per ml infusion	4	DF(S)	N-acetylcysteine 200 mg per ml concentrate for solution for infusion	2	DF(S + T)/PF
Morphine 20 mg sachet PR granules for oral suspension	4	DF(S)/PF	Suxamethonium chloride 100 mg per 2 ml injection	2	DF(S)/LP
Ranitidine 150 mg in 10 ml oral solution	4	EA/PF/LP	All other AiFs (OL) with frequency = 1	8	
Gabapentin 50 mg per ml SF oral solution	3	Pal/PF/LP	All other AiFs (UL) with frequency = 1	4	
Lansoprazole 15 mg orodispersible tablet	3	DF(S)/PF	Total number of AiFs identified	58	
Morphine sulphate 10 mg per 5 ml Oral solution	3	Pal/LP			
N-acetylcysteine 200 mg per ml concentrate for solution for infusion	3	DF(S + T)/PF			
Paracetamol 120 mg per 5 ml SF oral suspension	3	PF/LP			
Vigabatrin 500 mg sachet	3	DF(S)/PF			
Domperidone 1 mg per ml oral suspension	2	LP			
Glycopyrronium bromide 1 mg per 5 ml oral solution	2	LP			
Glycopyrronium bromide 1 mg tablet	2	DF(S + T)/PF			
Levothyroxine 50 mcg tablet	2	DF(T)/PF			
Lisinopril 2.5 mg Tablet	2	DF(T)/Pal			
Paracetamol 500 mg caplet	2	PP			
Phosphate 500 mg (16.1 mmol) effervescent tablet	2	DF(S)/PF			
Sildenafil 2.5 mg per ml oral suspension	2	PF/LP			
Sodium chloride 5 mmol per ml oral solution	2	EA/LP			
Vancomycin 500 mg powder for solution for infusion	2	LP			
All other AiFs (OL) with frequency = 1	35				
All other AiFs (UL) with frequency = 1	7				
Total number of AiFs identified	201				

All manufacturer/brand level details have been removed. Frequencies have been merged where the active pharmaceutical ingredient (API), strength and dosage form of the product identified were identical

DF(S), dosage form issue—strength; DF(T), dosage form issue—type; DF(S + T), dosage form issue—strength and type; PF, patient factors (swallow issue/enteral tube administration); Pal, palatability issue; LP, local policy (Standard practice manipulation); PP, patient preference; EA, Ease of Administration

## Discussion

### Statement of key findings

Age-appropriate assessment of DF administration has “context-independent” and “context-dependent” components. The AaF tool offers a structured approach to both components, recognises a multitude of reasons (including preference) and helps to minimise differences between assessors. Internal validation of the tool has demonstrated good IRR, albeit by the people who developed it.

The tool uses reports of direct observation of attempted/successful administration to confirm and thus expand upon the ages of children and reasons why a medicine may either be classified as AaF or AiF [23, 27–29]. The classification ‘Maybe age-appropriate’ indicates that the DF was either authorised but used OL or was an UL medicine. Further research and development by manufacturers and scrutiny by the regulator, or research and publication by scientists/clinicians, would give reassurance that such use is appropriate. When formulations are identified as being age-inappropriate, understanding the ‘real-life’ alternate administration strategies used by caregivers to improve acceptability, swallowability and/or dose adaptability of the product to facilitate administration to a child can be a vital source of information for the pharmaceutical industry and medicines regulators [30].

The need to develop new authorised medicines acceptable for children and their carers is important, as is development of paediatric versions of older medicines, some of which are UL [31–34]. Observational data collected in 2016 was used to develop and test the AaF tool in 2019. Since the AaF study patient level data was gathered, three medicinal products have been exclusively designed for use in children during 2016–2018 and approved under the EU Paediatric-Use Marketing Authorisation (PUMA); namely Glycopyrronium bromide (Sialanar®), Melatonin (Slenyto®) and Vigabatrin (Kigabeq®) [35]. Oral suspension of omeprazole was licensed in 2019 for children and adults [36, 37]. Inspection of our results shows that melatonin capsules and omeprazole MUPS frequently caused problems and were considered AiFs so these new authorised DFs might be expected to improve acceptability. Repeating observations of administration with the tool can confirm this. Anecdotally (NPPG online discussion forum https://nppg.org.uk/) suggests that issues still exist and that the additional cost of changing practice is a significant factor (e.g. Melatonin tablets continue to be crushed and administered with liquid rather than using Slenyto®).

The use of AiFs was identified across all age groups at both hospital sites, demonstrating a partial lack of suitable AaFs available for use in children at that time. The proportion of authorised medicines used was found to be higher at AH site, likely due to the age of the patient population as very few medicines have been specifically designed for neonatal use, thus younger children are more likely to encounter AiFs. The prevalence of ULOL medicine use in children, found to be 68.2% of MAEs (1,475/2,162) within this study, across both hospital sites, shows a significant unmet clinical need continues [38–41].

We have previously shown that DF modification at the point of administration to achieve the required dose is common in paediatric practice [42]. However, the safety and risks in terms of dose accuracy and handling associated with this practice are often unknown [11, 12]. This study demonstrated that manipulations of medicines used in children are performed for many reasons, including patient preference. The most common reason recorded in this study was due to an issue with the DF available, in terms of its strength and/or DF type.

Enteral tube administration (ETA) was identified as a common way to give medicines to children in an inpatient setting. The Summaries of Product Characteristics (SmPCs) for all oral medicines included in the study were reviewed for information in 2019, in relation to their use via any form of ETA. At AH, only 9% of 1,106 oral MAEs included information regarding administrations via a type of ETA. This figure was lower at LWH site, with only 5% of 206 oral MAEs mentioning use of ETA within the SmPC. Administering medicines through enteral tubes without appropriate information can potentially lead to inaccurate dosing and/or blockage of the feeding tube, especially when non-liquid medications are used [43]. Advice is provided on conducting appropriate studies [13, 44].

Other studies have reported assessment of AaFs [22, 23, 38, 45, 46] by screening lists of medicinal products according to set criteria or making assumptions for the expected ‘user’; in terms of generic age/weight band profiles and the anticipated physical capability of a child, consistent with paediatric developmental milestones. One such assumption is that children under 6 years are unable to swallow SODFs such as tablets, which is not the case [47]. Children, as young as 6 months [48] and even neonates [49], are capable of swallowing mini-tablets and for standard tablets this can be as young as 2 to 4 years, depending on the DF size [47, 50]. Participation in ‘pill-school’ education and training programmes is particularly helpful in enabling children to learn the skills necessary to swallow SODFs [51].

### Strengths and weaknesses

This study is one of few [39, 52] that includes individual patient/user information (including overall acceptability and palatability) which formed part of each medicine assessment; limiting the number of assumptions needed. In development it has expanded the detail in which AiFs can be categorised and subsequently, in application to the remaining MAEs it has been demonstrated to be capable of consistent application.

A limitation of this study could be the age of the observational data used to develop and validate the tool and to report on the use of AiFs at that time (2015–2016). The age of the data does not affect the internal validation. The components of the data collection were decided on before the tool was designed [53]. It is possible that other elements of data collection could lead to an improved tool. If practice changes it is simple to add additional reasons for manipulation of medicines if not covered adequately by the tool. Discussion with clinical practitioners, review of drugs in current practice and of current literature confirms that practice has changed little in the intervening time period and many of the useful improvements in paediatric formulation (e.g. mini-tablets; paediatric strength injections) have not yet been placed before regulators or brought to market [54].

Not all aspects of a formulation contributing to age-appropriateness were assessed (e.g. excipient content) [55–57]. The proportion of medicines found to be age-appropriate may be an overestimate particularly for oral liquids [55]. A simple question can be added to the tool to accommodate information on the suitability of excipients. No ‘unsuccessful’ administrations were recorded as part of this study. This may relate to the specialist hospital settings and the “one-day snapshot” study design as problematic medicines may have been resolved, prior to review.

### Interpretation and further research

There is a need to quantify the impact of using AiFs for carers/patients, in terms of their safety, inconvenience and cost (using a risk–benefit analysis) [58, 59]. External validation should be undertaken in differing settings and the tool adapted for those prescribing and selecting appropriate medicines to administer.

## Conclusion

A tool has been developed and validated to distinguish AaFs and AiFs for children from neonates to adolescents taking account of actual medicine administration practice. The data generated have identified areas for future paediatric drug development. The tool has shown good IRR, but further work is required to externally validate and assess the tool’s utility within different settings.

## Supplementary Information

Below is the link to the electronic supplementary material.

**Online Resource 1.** Age-appropriate assessment outcome categories (PDF 148 kb)

**Online Resource 2**. % of AaFs v AiFs identified across age groups at AH and LWH (PDF 150 kb)
