# Supplementary material for: Development and evaluation of an assessment of the age-appropriateness/inappropriateness of formulations used in children
Source: Int J Clin Pharm. 2022 Oct 8;44(6):1394–405. doi: 10.1007/s11096-022-01478-5 (PMC9718882; doi:10.1007/s11096-022-01478-5)
Supplement: Supplementary file 1 — Online Resource 1. Age-appropriate assessment outcome categories (PDF 148 kb) [file 11096_2022_1478_MOESM1_ESM.pdf]

SUPPLEMENTARY DATA: ONLINE RESOURCE 1

Development and preliminary evaluation of an assessment of the age-appropriateness/age-inappropriateness of formulations used in children

International Journal of Clinical Pharmacy (IJCP)

Jennifer C. Duncan[1*], Louise E. Bracken[1], Anthony J. Nunn[1,2], Matthew Peak[1,2], Mark A. Turner[1,2,3]
*Corresponding Author: Jennifer.Duncan@alderhey.nhs.uk

1 Paediatric Medicines Research Unit, Institute in the Park, Alder Hey Children's NHS Foundation Trust, Liverpool, United Kingdom 2 Department of Women's & Children's Health, Institute of Translational Medicine, University of Liverpool, Liverpool Health Partners, Liverpool, United Kingdom 3 Liverpool Women's NHS Foundation Trust, Liverpool, United Kingdom.

Table S1. Age-appropriate assessment outcome categories

Top Level Assessment Outcome	AaF Assessment Code
**Product Unassessable**	**U**
Medical Device (includes any manipulations needed)	MD
Age-Appropriate Formulation (including permitted manipulations)	AaF
Maybe Age-Appropriate Formulation (Off-label including permitted manipulations)	M (OL)
Maybe Age-Appropriate Formulation (Unlicensed including permitted manipulations)	M (UL)
Age-inappropriate Formulation (Off-label with unauthorised manipulations)	AiF (OL)
Age-inappropriate Formulation (Unlicensed with unauthorised manipulations)	AiF (UL)

Table S2. Detailed age-appropriate assessment outcome sub-categories

Detailed Assessment Outcome Result
**Product Unassessable (U) - Manufacturer Unknown**
**Product Unassessable (U) - Missing Information**
Product Unassessable (U) - Other (Specify) >>> SEND TO PANEL
Medical Device (MD) - Suitable Product (No issues)
Medical Device (MD) - Product Required Manipulation
Medical Device (MD) - Other (Specify) >>> SEND TO PANEL
AaF - Authorised Medicine (Paediatric Indication covered by MA)
AaF - Authorised Medicine (Paediatric Indication covered by MA using Permitted Manipulation)
AaF - Authorised Medicine (OK once issue resolved + Paediatric Indication covered by MA)
AaF - Authorised Medicine (OK once issue resolved + Paediatric Indication covered by MA using Permitted Manipulation)
M (OL) - DF OK / No Manipulation Required
M (OL) - DF OK / Permitted Manipulation Used *(Record source used - Manufacturer only)*
M (OL) - DF OK / Added to Milk Feed (Neonate/Infants)
M (OL) - Other (Specify) >>> SEND TO PANEL
M (UL) - DF OK / No Manipulation Required
M (UL) - DF OK / Permitted Manipulation Used *(Record source used - Manufacturer only)*
M (UL) - DF OK / Added to Milk Feed (Neonate/Infants)
M (UL) - Other (Specify) >>> SEND TO PANEL

Detailed Assessment Outcome Result
AiF (OL) - DF Issue
AiF (OL) - DF Issue + Added to Milk Feed (Neonate/Infants)
AiF (OL) - DF Issue (via Enteral Tube)
AiF (OL) - DF Issue (via Enteral Tube) + Added to Milk Feed (Neonate/Infants)
AiF (OL) - DF Issue + Added to Food (Ease of Administration)
AiF (OL) - DF Issue + Added to Food (Palatability Issue / Taste Masking)
AiF (OL) - DF Issue + Added to Food (Reason Unknown)
AiF (OL) - DF Issue + Added to Drink (Ease of Administration)
AiF (OL) - DF Issue + Added to Drink (Palatability Issue / Taste Masking)
AiF (OL) - DF Issue + Added to Drink (Reason Unknown)
AiF (OL) - DF Issue + Addition to Thickener (Swallowing Issue)
AiF (OL) - DF Issue + Addition to Thickener (Swallowing Issue) + Added to Drink (Ease of administration)
AiF (OL) - Patient Preference
AiF (OL) - Medicine Refused
AiF (OL) - Added to Food (Ease of Administration)
AiF (OL) - Added to Food (Palatability Issue / Taste Masking)
AiF (OL) - Added to Food (Reason Unknown)
AiF (OL) - Added to Drink (Ease of Administration)
AiF (OL) - Added to Drink (Palatability Issue / Taste Masking)
AiF (OL) - Added to Drink (Reason Unknown)
AiF (OL) - Mixed with other medicine(s)
AiF (OL) - Addition to Thickener (Swallowing Issue)
AiF (OL) - Convenience
AiF (OL) - Compliance / Adherence Issue
AiF (OL) - Standard Practice / Policy Manipulation
AiF (OL) - Other (Specify) >>> SEND TO PANEL
AiF (UL) - DF Issue
AiF (UL) - DF Issue + Added to Milk Feed (Neonate/Infants)
AiF (UL) - DF Issue (via Enteral Tube)
AiF (UL) - DF Issue (via Enteral Tube) + Added to Milk Feed (Neonate/Infants)
AiF (UL) - DF Issue + Added to Food (Ease of Administration)
AiF (UL) - DF Issue + Added to Food (Palatability Issue / Taste Masking)
AiF (UL) - DF Issue + Added to Food (Reason Unknown)
AiF (UL) - DF Issue + Added to Drink (Ease of Administration)
AiF (UL) - DF Issue + Added to Drink (Palatability Issue / Taste Masking)
AiF (UL) - DF Issue + Added to Drink (Reason Unknown)
AiF (UL) - DF Issue + Addition to Thickener (Swallowing Issue)
AiF (UL) - DF Issue + Addition to Thickener (Swallowing Issue) + Added to Drink (Ease of administration)
AiF (UL) - Patient Preference
AiF (UL) - Medicine Refused
AiF (UL) - Added to Food (Ease of Administration)
AiF (UL) - Added to Food (Palatability Issue / Taste Masking)
AiF (UL) - Added to Food (Reason Unknown)

Detailed Assessment Outcome Result
AiF (UL) - Added to Drink (Ease of Administration)
AiF (UL) - Added to Drink (Palatability Issue / Taste Masking)
AiF (UL) - Added to Drink (Reason Unknown)
AiF (UL) - Mixed with Other Medicine(s)
AiF (UL) - Addition to Thickener (Swallowing Issue)
AiF (UL) - Convenience
AiF (UL) - Compliance / Adherence Issue
AiF (UL) - Standard Practice / Policy Manipulation
AiF (UL) - Other (Specify) >>> SEND TO PANEL
UNCLEAR - Send to Panel (For Discussion)
