# Supplementary material for: Development and evaluation of an assessment of the age-appropriateness/inappropriateness of formulations used in children
Source: Int J Clin Pharm. 2022 Oct 8;44(6):1394–405. doi: 10.1007/s11096-022-01478-5 (PMC9718882; doi:10.1007/s11096-022-01478-5)
Supplement: Supplementary file 2 — Online Resource 2. % of AaFs v AiFs identified across age groups at AH and LWH (PDF 150 kb) [file 11096_2022_1478_MOESM2_ESM.pdf]

SUPPLEMENTARY DATA: ONLINE RESOURCE 2

Development and preliminary evaluation of an assessment of the age-appropriateness/age-inappropriateness of formulations used in children

International Journal of Clinical Pharmacy (IJCP)

Jennifer C. Duncan[1*], Louise E. Bracken[1], Anthony J. Nunn[1,2], Matthew Peak[1,2], Mark A. Turner[1,2,3]
*Corresponding Author: Jennifer.Duncan@alderhey.nhs.uk

1 Paediatric Medicines Research Unit, Institute in the Park, Alder Hey Children's NHS Foundation Trust, Liverpool, United Kingdom 2 Department of Women's & Children's Health, Institute of Translational Medicine, University of Liverpool, Liverpool Health Partners, Liverpool, United Kingdom 3 Liverpool Women's NHS Foundation Trust, Liverpool, United Kingdom.

Figure S3. % of AaFs v AiFs identified across age groups at AH

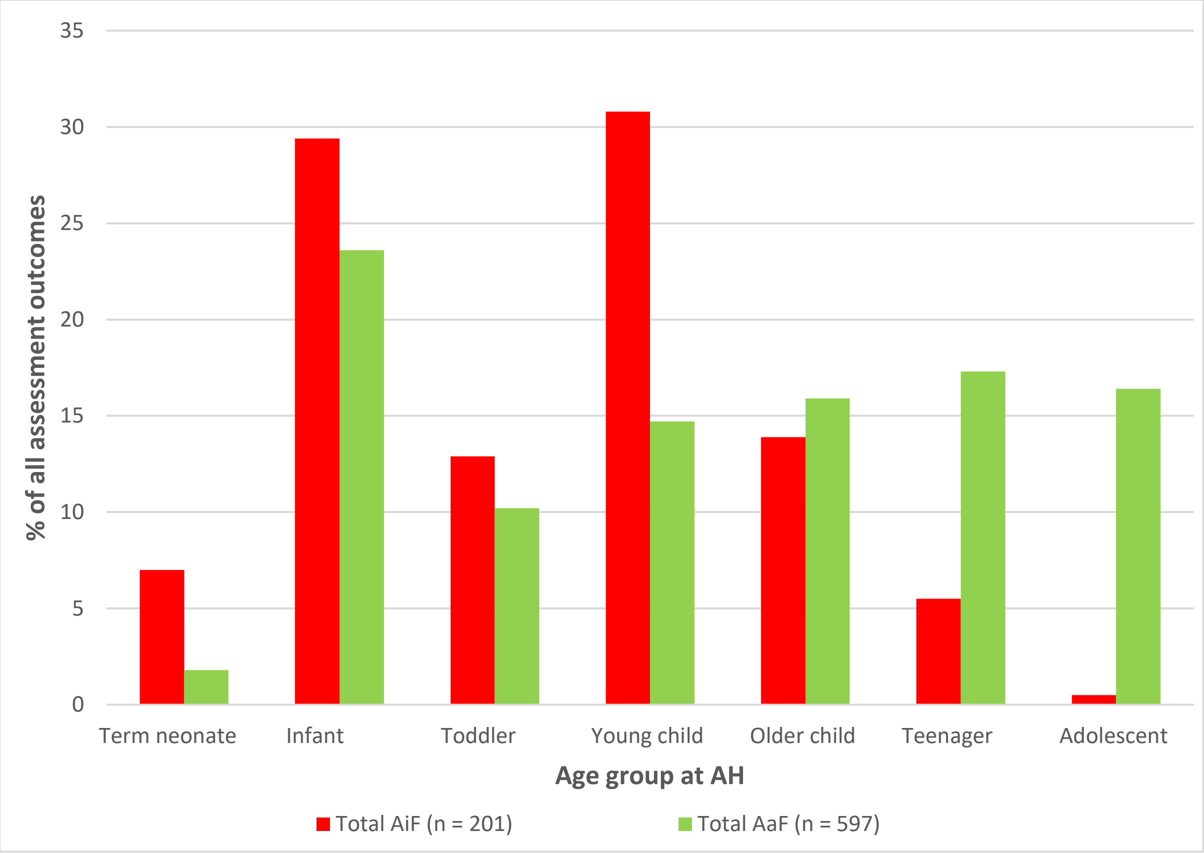

Figure S4. % of AaFs v AiFs identified across age groups at LWH

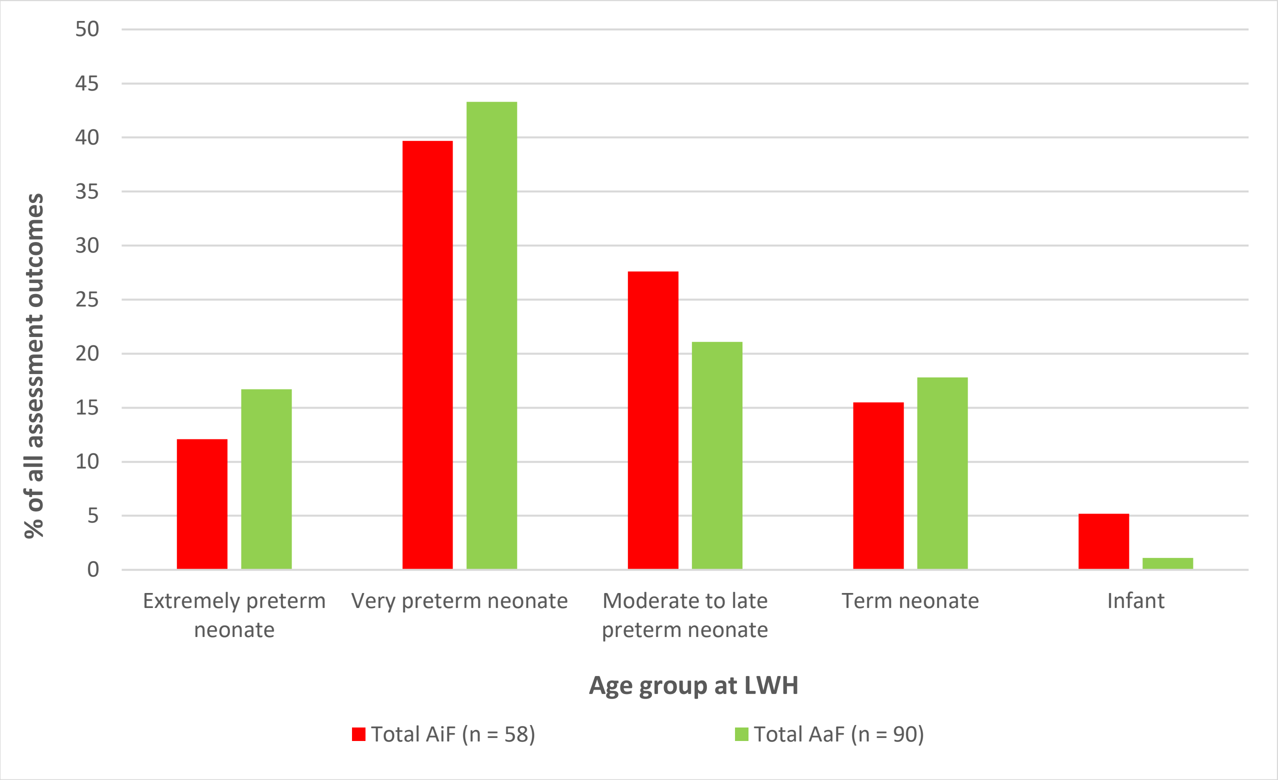

Table S5. Final assessment outcome results across the age groups at AH (n = 1,758 MAEs)

AaF Assessment Code	Age group							Total (N)
	Term neonate (as % of n)	Infant (as % of n)	Toddler (as % of n)	Young child (as % of n)	Older child (as % of n)	Teenager (as % of n)	Adolescent (as % of n)	
**Number of Patients**	37	102	33	60	49	35	18	334
AaF	11 (10.4)	141 (29.7)	61 (30.2)	88 (24.4)	95 (37.0)	103 (50.0)	98 (64.1)	597 (34.0)
AiF (OL)	14 (13.2)	47 (9.9)	21 (10.4)	39 (10.8)	20 (7.8)	9 (4.4)	1 (0.7)	151 (8.6)
AiF (UL)		12 (2.5)	5 (2.5)	23 (6.4)	8 (3.1)	2 (1.0)		50 (2.8)
M (OL)	50 (47.2)	179 (37.8)	90 (44.6)	143 (39.7)	94 (36.6)	65 (31.6)	28 (18.3)	649 (36.9)
M (UL)	30 (28.3)	85 (17.9)	22 (10.9)	64 (17.8)	30 (11.7)	24 (11.7)	21 (13.7)	276 (15.7)
MD		6 (1.3)	3 (1.5)	1 (0.3)	5 (1.9)	3 (1.5)	3 (2.0)	21 (1.2)
U	1 (0.9)	4 (0.8)		2 (0.6)	5 (1.9)		2 (1.3)	14 (0.8)
**Total MAEs (n)**	**106**	**474**	**202**	**360**	**257**	**206**	**153**	**1758**
**Total AiF (as % of N)**	**14 (7.0)**	**59 (29.4)**	**26 (12.9)**	**62 (30.8)**	**28 (13.9)**	**11 (5.5)**	**1 (0.5)**	**201**
**Total AaF (as % of N)**	**11 (1.8)**	**141 (23.6)**	**61 (10.2)**	**88 (14.7)**	**95 (15.9)**	**103 (17.3)**	**98 (16.4)**	**597**

Table S6. Final assessment outcome results across the age groups at LWH (n = 441 MAEs)

AaF Assessment Code	Age group					Total (N)
	Extremely preterm neonate (as % of n)	Very preterm neonate (as % of n)	Moderate to late preterm neonate (as % of n)	Term neonate (as % of n)	Infant (as % of n)	
**Number of Patients**	8	31	38	14	2	93
AaF	15 (34.9)	39 (24.4)	19 (12.6)	16 (24.2)	1 (4.8)	90 (20.4)
AiF (OL)	7 (16.3)	23 (14.4)	12 (7.9)	9 (13.6)	3 (14.3)	54 (12.2)
AiF (UL)			4 (2.6)			4 (0.9)
M (OL)	16(37.2)	89 (55.6)	97 (64.2)	30 (45.5)	10 (47.6)	242 (54.9)
M (UL)	5 (11.6)	7 (4.4)	19 (12.6)	11 (16.7)	7 (33.3)	49 (11.1)
MD						0 (0.0)
U		2 (1.3)				2 (0.5)
**Total MAEs (n)**	**43**	**160**	**151**	**66**	**21**	**441**
**Total AiF (as % of N)**	**7 (12.1)**	**23 (39.7)**	**16 (27.6)**	**9 (15.5)**	**3 (5.2)**	**58**
**Total AaF (as % of N)**	**15 (16.7)**	**39 (43.3)**	**19 (21.1)**	**16 (17.8)**	**1 (1.1)**	**90**
